# Hurthle Cell Thyroid Carcinoma with Liver and Paraaortic Abdominal Nodal Metastasis: Progression on Sorafenib Therapy after Initial Disease Stabilization

**DOI:** 10.1055/s-0042-1759615

**Published:** 2022-12-20

**Authors:** Sarvesh Loharkar, Sandip Basu

**Affiliations:** 1 Radiation Medicine Centre, Bhabha Atomic Research Centre, Tata Memorial Hospital Annexe, Parel, Mumbai, Maharashtra, India; 2 Homi Bhabha National Institute, Mumbai, Maharashtra, India

**Keywords:** Hurthle cell thyroid carcinoma, ^18^ F-FDG-PET/CT, tyrosine kinase inhibitors, para-arotic lymphadenopathy, sorafenib, lenvatinib, radioiodine-refractory

## Abstract

Hurthle cell thyroid carcinoma (HCTC) demonstrates inferior prognosis compared with other types of differentiated thyroid cancer (DTC), along with radioiodine refractoriness and relatively poor ^131^ I concentrating ability. We herein report a case of a middle-aged lady presenting with neck swelling for years, who on pre-surgery work-up was diagnosed to harbor metastatic nodal and lung lesions. Post-thyroidectomy and neck dissection, she was diagnosed with HCTC. Post-surgery, none of the lesions concentrated radioactive-iodine (RAI) sufficiently but showed FDG avid lesions as mediastinal nodes, lung nodules, solitary lytic sternal lesions, and unusual bilateral paraaortic abdominal nodes. She was put on tyrosine kinase inhibitor (sorafenib) and showed disease stabilization for the initial 3 years, but multiple toxicity symptoms while on sorafenib therapy that needed multiple dose adjustments. Over the period of the subsequent year, she developed significant disease progression with liver involvement. She was shifted to lenvatinib, which she tolerated well. The functional imaging profile with unusual metastatic sites, the aggressive clinical presentation and disease course of RAI refractory HCTC over 4 years on tyrosine kinase inhibitor therapy, and the role of molecular FDG-PET/CT imaging in disease monitoring and clinical management of such case is presented.

## Introduction

HCTC, earlier designated as a rare subtype of follicular thyroid carcinoma (FTC), is now classified it as a distinct tumor type by the 2017 WHO classification owing to significant histopathological and molecular differences with FTC. It is estimated to comprise 3 to 7% of DTCs. 1 The histopathological examination of HCTC shows the predominance of the characteristic oncocytic cells. Multiple large series have proven its adverse prognosis compared with other forms of DTCs, especially papillary thyroid cancer, and more recurrences and poorer survival rates than pure FTC population. The lesser fraction of these tumors (estimated at 10% of HCTC metastases) concentrates RAI. Compared with FTCs these commonly involve regional lymph nodes while also showing a greater propensity for distant metastases. 2 Thus, HCTC patients may present with symptomatic metastases and adequate preoperative staging using a cross-sectional imaging (CT scan) is often advocated. After surgery, RAI ablation therapy is considered next-line management and in metastatic disease, it has shown improved survival rates in RAI concentrating tumors. For RAI refractory thyroid cancer, especially in grossly metastatic and symptomatic cases, the tyrosine kinase inhibitors (TKIs) form a mainstay of treatment, including HCTCs. TKIs stabilize the disease and prolong PFS; however, bear multiple toxic effects and have limited long-term follow-up data at present. 3 Herein, we present a patient of HCTC with RAI-refractory features and FDG-avid distance metastases. Unusual paraortic nodal and liver metastases and progression after initial 3 years of disease stabilization on TKI therapy (sorafenib) are other findings noteworthy in the case.

## Case Report

**Fig. 1:**
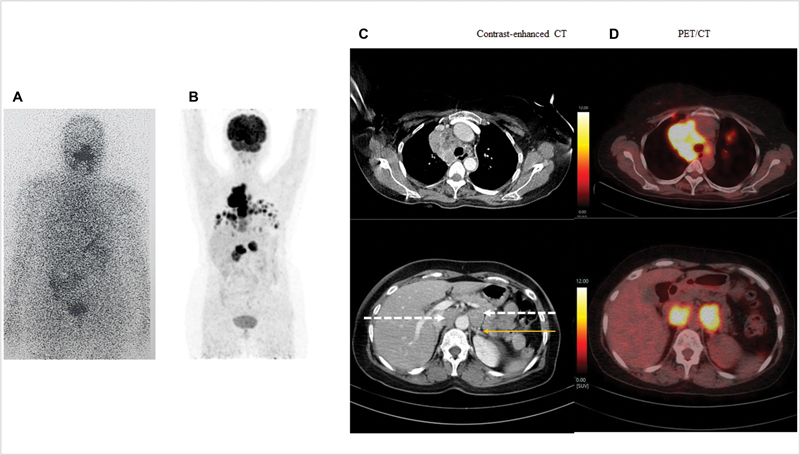
( **A** ) Baseline radioiodine planar gamma camera scan showing diffuse low-grade uptake in chest region. ( **B** ) ^18^ F-FDG PET/CT (MIP image) post-thyroidectomy showing metabolically active multiple mediastinal nodal masses, lung nodules, and bilateral abdominal soft tissue density masses, which on ( **C** ) shows contrast-enhanced CT scan labeled as paraaortic nodal masses ( *dotted arrow* ) adjacent to adrenal glands ( *yellow solid arrow* ).

A 56-years-old female patient presented with a large neck mass that gradually increased in size over the 8 years. The USG-guided FNAC from thyroid tissue turned out to be of Bethesda category IV. She underwent a staging CT scan that revealed a large heterogeneously enhancing lesion in the left lobe of thyroid, multiple metastatic pulmonary nodules, and metastatic mediastinal nodes were also noted. She underwent total thyroidectomy, bilateral central compartment, and selective neck dissection along with mediastinoscopy, the histopathology was suggestive of Hurthle cell variant of differentiated thyroid cancer (HCTC), with gross extrathyroidal extension and metastasis to left sided neck nodes. A biopsy from the largest right sided lung nodule turned out as metastatic thyroid carcinoma with Hurthle cell change. She underwent an RAI scan ( Fig. 1A ) that showed low-grade uptake in the chest region slightly above background only followed by high-dose RAI therapy with 181 mCi dose. The post-therapy scan also showed very low-grade uptake in the chest region; a non–contrast-enhanced ^18^ F-FDG-PET/CT ( Figs. 1B, D ) revealed FDG avid bilateral lung nodules, mediastinal nodal mass, and bilateral abdominal soft tissue lesions and solitary lytic bone lesion in the sternum. Initially, these abdominal masses were suspected of bilateral adrenal metastases but contrast-enhanced CT undertaken later confirmed these to be para-aortic nodal masses ( Fig. 1C, D ). The repeat whole body scan using ^131^ I at 6 months following ^131^ I treatment demonstrated no iodine avid focus in the whole body survey, with stimulated serum Tg level over 300 ng/mL.

**Fig. 2:**
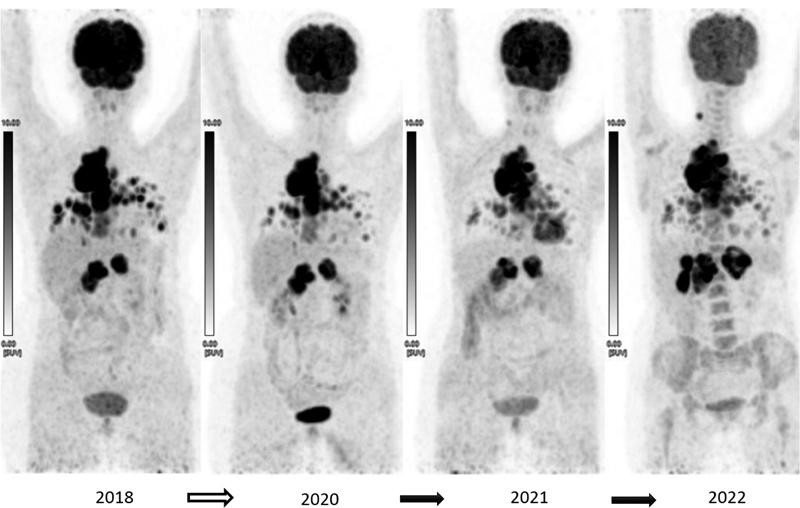
Serial ^18^ F-FDG PET/CT MIP images showing initial stabilization of lesions for 3 years up to 2021, which progressed further in 2022 when there was increase in the size and metabolism of mediastinal and abdominal nodal masses and newly seen liver lesion.

**Fig. 3:**
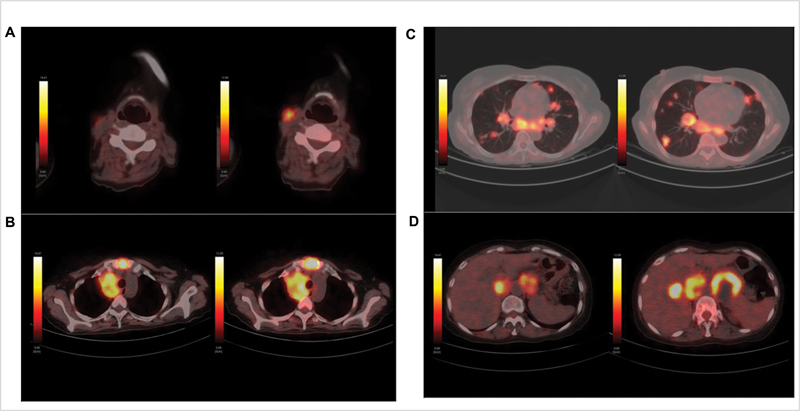
^18^ F-FDG-PET/CT fused transaxial images (scan done in 2021 on *left* and in 2022 on *right* side) showing increase in size and FDG uptake of ( **A** ) left-sided cervical level III node, ( **B** ) lytic sternal lesion and mediastinal nodal mass, ( **C** ) multiple lung nodules in bilateral lungs, and ( **D** ) bilateral para-aortic nodal masses with newly seen liver lesion.

Considering her symptomatic status for breathing difficulties and RAI-refractory status she was started on tablet Sorafenib 400 mg twice daily, the dose reduced within a month of treatment initiation in view of grade 3 hand-foot syndrome (HFS) and diarrhea, to 200 mg twice daily for 6 months and subsequently gradually escalated to 400 mg twice daily in next 6 months. Post-1-year response evaluation PET/CT showed stable disease and a 50% decrease in her symptoms and she continued sorafenib for further 2 years with no significant toxicity and progression ( Fig. 2 ). The 3 year treatment response evaluation ^18^ F-FDG-PET/CT showed a new right-sided tiny cervical lesion, some increase in FDG uptake, size, and number of pulmonary nodules, mediastinal nodes, sternal lesion, though some decrease in FDG uptake of para-aortic nodes was noted. Considering the COVID-19 pandemic and patient-related logistic issues, multidisciplinary team decided to continue the same regimen of TKI. Again, she developed grade-2 HFS, hypertension, and low platelet counts, for which a lower dose regimen was titrated. The following response evaluation ^18^ F-FDG-PET/CT demonstrated disease progression with new FDG-avid liver lesion and increase in the FDG uptake, size, and number of other lesions (lung nodules, mediastinal and abdominal nodes, lytic sternal lesion) ( Figs. 2 and 3 ). The serum thyroglobulin (Tg) value (with suppressed TSH) on all occasions were more than 300 ng/mL. Additionally, the patient complained of pyrexia of unknown origin for 1 month for which all serological, microbiological workup and ^18^ F-FDG PET/CT did not reveal any obvious cause and was suspected as disease-related fever. Meanwhile, she underwent next-generation sequencing (NGS) for RET, NTRK, ALK, BRAF, etc, for any actionable mutations, but revealed only positive Tier III (variant of unknown significance) missense mutation in exon 3 of the *HRAS* gene. She was switched over to tablet lenvatinib 18 mg, which she is tolerating well at present and recorded a reduction in episodes of fever spikes also.

## Discussion

Long-standing palpable neck mass is the most common presentation for HCTCs followed by pressure symptoms such as dyspnea, both of them were the only symptoms present in our case. Residual tumors after surgery and the presence of distant metastases are considered important prognostic factors. Among distant metastases, the lung is most commonly reported followed by bone and mediastinum. 4 Similar was followed in our case but the liver and abdominal nodal metastases are rare. Only a few such reports exist in the literature: (i) Meyer et al 5 reported pelvic nodal mass in insular carcinoma, while (ii) anaplastic transformation of papillary thyroid cancer in form of mesenteric mass was reported by Hosoda et al 6 ; but our case showed the unique observation of bilateral paraaortic nodal metastases in HCTC. As noted in this case, lack of adequate RAI concentration, a common observation in HCTCs precludes the use of powerful RAI therapy. ^18^ F-FDG-PET/CT has proven its added role over RAI scan and CECT in assessing metastatic disease, especially in cases with low RAI concentration. Furthermore, such as other tumors, high-grade ^18^ F-FDG uptake is considered an indicator of poor prognosis. Similar was noted in our case as she showed almost no RAI concentration and high FDG concentration in metastatic lesions eventually progressed despite all standard of care. It is imperative that all cases of metastatic HCTC or with high-serum thyroglobulin level, irrespective of RAI concentration status at baseline be evaluated using ^18^ F-FDG-PET/CT. 7

In radioiodine refractory thyroid carcinoma, systemic therapy with TKIs has currently evolved as the treatment of choice. Though improved survival has been documented both in retrospective and prospective data, adverse effects are a major concern with their use. Even our case showed multiple symptoms such as HFS, diarrhea, thrombocytopenia, and hypertension with sorafenib but almost all were manageable with dose modifications. Our patient showed an initial response (disease stabilization) for 3 years on sorafenib but progressed subsequently. Also reported initial flare phenomenon and rapid progression after discontinuing TKIs. 8 Many groups such as Aydemirli et al have highlighted the utility of genomic sequencing and using targeted treatments too in HCTC. 9 This was followed in our case but remained unsuccessful due to lack of targetable mutation.

## Conclusion

HCTCs are aggressive and can be commonly observed as RAI refractory and metastatic. ^18^ F-FDG PET/CT is a promising imaging modality for staging, disease burden assessment, and also for treatment assessment, especially in systemic metastatic disease. TKI though currently considered a mainstay in metastatic and symptomatic RAI-refractory thyroid cancer patients, should be used cautiously considering their multiple toxicities and monitored for long-term disease control.
